# Dose-Dependent Effect of Tilmicosin Residues on *erm*A Rebound Mediated by *IntI*1 in Pig Manure Compost

**DOI:** 10.3390/microorganisms13092123

**Published:** 2025-09-11

**Authors:** Pengfei Zhang, Qingnan Mo, Chang Liu, Qing Liu, Jiaojiao Xu, Yan Wang, Xin Wen, Yinbao Wu

**Affiliations:** 1 National Engineering Research Center for Breeding Swine Industry, College of Animal Science, South China Agricultural University, Guangzhou 510642, China; scauzpf@163.com (P.Z.); moqingnan2021@163.com (Q.M.); liuchang210509@163.com (C.L.); liuqing970317@126.com (Q.L.); xujiaojiao98@foxmail.com (J.X.); ywang@scau.edu.cn (Y.W.); 2 Guangdong Provincial Key Laboratory of Agro-Animal Genomics and Molecular Breeding, South China Agricultural University, Guangzhou 510642, China; 3 State Key Laboratory of Swine and Poultry Breeding Industry, South China Agricultural University, Guangzhou 510642, China; 4 Key Laboratory of Chicken Genetics, Breeding and Reproduction, Ministry of Agriculture, South China Agricultural University, Guangzhou 510642, China; 5 Ministry of Agriculture Key Laboratory of Tropical Agricultural Environment, South China Agricultural University, Guangzhou 510642, China

**Keywords:** tilmicosin, pig manure, composting, macrolide resistance genes, horizontal gene transfer, bacteria

## Abstract

The impact of varying antibiotic residue levels on antibiotic resistance gene (ARG) removal during composting is still unclear. This study investigated the impact of different residue levels of tilmicosin (TIM), a common veterinary macrolide antibiotic, on ARG removal during pig manure composting. Three groups were used: the CK group (no TIM), the L group (246.49 ± 22.83 mg/kg TIM), and the H group (529.99 ± 16.15 mg/kg TIM). Composting removed most targeted macrolide resistance genes (MRGs) like *ere*A, *erm*C, and *erm*F (>90% removal), and reduced *erm*B, *erm*X, *erm*Q, *acr*A, *acr*B, and *mef*A (30–70% removal). However, *erm*A increased in abundance. TIM altered compost community structure, driving succession through a deterministic process. At low doses, TIM reduced MRG–bacteria co-occurrence, with horizontal gene transfer via *intI*1 being the main cause of *erm*A rebound. In conclusion, composting reduces many MRG levels in pig manure, but the persistence and rebound of genes like *erm*A reveal the complex interactions between composting conditions and microbial gene transfer.

## 1. Introduction

The rise of antibiotic resistance and drug-resistant infections is a major public health issue. Internationally, there is consensus that antibiotics must be used more carefully in human medicine and animal production [1,2]. More than half of antibiotics in China (52%) are used in livestock production, raising concerns that residual traces may accelerate resistance [3]. Despite a 2020 ban on growth-promoting antibiotics, therapeutic antibiotics still result in residues and antibiotic resistance genes (ARGs) in animal feces [4]. Approximately 58% of these antibiotics are discharged into ecosystems through livestock excreta [5]. Applying animal manure as fertilizer enhances soil fertility while reintroducing ARGs from treated animals into the environment [6,7].

Aerobic composting offers an environmentally sustainable and economical approach to livestock waste treatment, effectively decreasing ARBs (antibiotic-resistant bacteria)/ARGs while generating safer fertilizers [8,9,10]. High temperatures in composting kill pathogenic bacteria and drug-resistant microorganisms while degrading most antibiotics in manure [11,12]. Composting also reduces antibiotic and resistance gene contamination by altering microbial community composition, affecting ARGs’ diversity and abundance [13]. Aerobic composting significantly decreases ARGs and mobile genetic elements (MGEs). Zhou et al. [14] found that over 90% of ARGs and MGEs were eliminated during chicken manure composting. Similarly, Zhang et al. [10] reported a significant reduction (83%) in ARGs after two weeks of composting cattle manure. However, some ARGs may remain unchanged or increase, as seen with *tet*C, *tet*X, *sul*1, and *sul*2 in cow manure [15]. Johnson et al. [16] observed varying outcomes for resistance gene clusters in different farm composts, highlighting the need to understand the factors driving these differences.

The fate of ARGs throughout the composting process is influenced by multiple interacting parameters, including composting material, temperature, pH, carbon-to-nitrogen ratio, and bacterial flora evolution [17]. Research has indicated the crucial involvement of microbiota throughout aerobic treatment processes [18], where ARBs serve as primary vectors for ARG dissemination through horizontal gene transfer (HGT) and vertical gene transfer (VGT), effectively determining ARGs’ fate in composting scenarios [17]. MGEs emerge as master regulators of ARG dissemination through horizontal transfer cascades [19]. Therefore, it is necessary to understand the fate of different ARGs in different environments as well as to analyze the influencing factors.

Tilmicosin (TIM), a semisynthetic macrolide antimicrobial antibiotic, is extensively used for prophylactic and therapeutic purposes against bacterial pneumonia and respiratory diseases induced by pathogens including *Pasteurella*, *Staphylococcus*, and *Streptococcus* [20]. Tilmicosin has received regulatory approval in the United States, China, Australia, and Italy for use in preventing and controlling susceptible bacterial infections in swine. It is particularly indicated in the management of respiratory diseases in pigs, including porcine pleuropneumonia and pasteurellosis [21]. In the United States, the approved daily dosage of tilmicosin for the prevention or treatment of diseases in pigs ranges from 200 to 400 mg per kilogram of body weight [22]. Here, we composted swine manure containing differential TIM concentrations to analyze the elimination patterns and key determinants of macrolide resistance genes (MRGs). The results comprehensively elucidate the diversity and abundance profiles of ARGs during livestock manure composting, providing critical insights for assessing and mitigating associated public health risks from animal manure and compost.

## 2. Materials and Methods

### 2.1. Sample Collection

**Figure 1:**
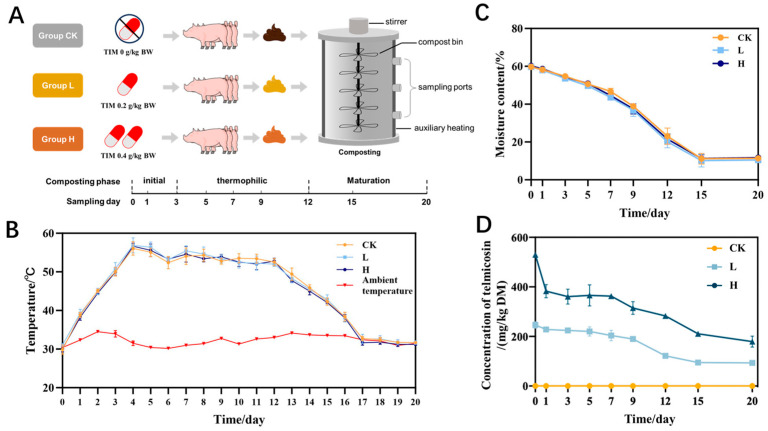
Composting process monitoring: (**A**) system design, (**B**) moisture content, (**C**) temperature changes, (**D**) TIM levels. Groups: CK (control), L (246.49 ± 22.83 mg/kg), H (529.99 ± 16.15 mg/kg).

Thirty commercial Duroc × Landrace × Yorkshire (DLY) barrows (a standard three-way cross breed) with an average weight of 32.10 ± 3.15 kg and without any history of antibiotic use for treatment and prevention during the previous month were selected for the pig manure collection experiment. A total of 30 pigs were randomly divided into three groups: the blank control group (CK group), low-concentration group (L group) and high-concentration group (H group). The CK group was fed only the basic diet. According to the 2015 edition of the Chinese Veterinary Drug Code and the feed intake of experimental pigs, the experimental pigs in the low-concentration group and the high-concentration group were fed the lowest and highest therapeutic doses of TIM, namely, 200 g/1000 kg feed and 400 g/1000 kg feed, respectively, which corresponded to 1.8 g/day and 3.6 g/day, respectively (Figure 1A). The animals were fed daily at 8 am and treated for 15 consecutive days with a 14-day rest period. The prefeeding period occurred one week before the formal collection of pig manure. During the prefeeding period, no animal antibiotics were used, and the experimental pigs presented no clinical abnormalities before entering the formal collection period. In accordance with the excretion rule for TIM in pig manure, manure was collected twice daily at 9 AM and 5 PM during the 15-day treatment period for composting experiments. Then, 200 g aliquots were collected vertically (top/center/base) during 20-day composting (sampling days: 0, 1, 3, 5, 7, 9, 12, 15, 20), and the collected pig manure was stored in a 4 °C refrigerator for subsequent composting experiments and measurement of indicators.

### 2.2. Composting Experiment

Supplementary Table S1 characterizes the raw substrates. Each experimental group was initiated with 8 kg of substrate material and conducted in three independent replicates. All composting trials were carried out at South China Agricultural University (Guangzhou, China) using 19 L cylindrical reactors certified by Chinese Patent No. ZL201520036620.0. The 25-day experimental phase incorporated intermittent aeration (140 L/min for a 10 min duration within 30 min cycles) coupled with daily mechanical turning (10 min at 16:00). The composting system maintained precise temperature control through three consecutive phases: (1) mesophilic adaptation at 35 ± 0.5 °C during the initial 24 h period, (2) transition to 45 ± 0.5 °C for the subsequent 24 h to initiate thermophilic conditions, and (3) sustained thermophilic operation at 55 ± 0.5 °C for 144 h (days 3–8). Supplemental heating was discontinued thereafter, allowing natural temperature progression through the maturation phase (days 9–25).

### 2.3. Measurement of Indicators

The study involved regular monitoring of multiple physicochemical parameters. Temperature was recorded three times daily (08:30, 12:30 and 16:30), while pH and electrical conductivity (EC) were measured using a pH meter and conductivity meter, respectively. Moisture content, total nitrogen (TN), ammonium nitrogen (${\mathrm{NH}}_{4}^{+}$-N), nitrate nitrogen (${\mathrm{NO}}_{3}^{-}$-N), and total organic carbon (TOC) were analyzed to determine the C/N ratio. TN and TOC were quantified via the Kjeldahl method and potassium dichromate oxidation, respectively. For ${\mathrm{NH}}_{4}^{+}$-N and ${\mathrm{NO}}_{3}^{-}$-N, extraction with potassium chloride followed by distillation titration was employed, with ${\mathrm{NO}}_{3}^{-}$-N additionally requiring ferrous sulfate reduction, as per Lu [23]. All measurements were based on dry weight corrections, with sample masses precisely recorded during analysis.

### 2.4. Determination of the Tilmicosin Concentration

For each compost sample collected at different time points (days 0, 1, 3, 5, 7, 9, 12, 15 and 20), 1.0 g aliquots were processed using an extraction protocol. Samples were mixed with 7.5 mL of 0.1 mol/L Na_2_EDTA Mcllvaine-methanol solution (2:1 buffer/methanol ratio) and subjected to vortex mixing followed by ultrasonication. After high-speed centrifugation, the supernatant was collected. This extraction procedure was performed twice for each sample, with combined supernatants subsequently purified through Oasis HLB columns (Waters Corp., Milford, MA, USA). Column elution was carried out using methanol containing 2% formic acid. The final extracts were filtered through 0.22 μm membranes prior to LC-MS analysis (Agilent Technologies, Santa Clara, CA, USA). TIM extraction and quantification followed established methods [24], with the analytical method demonstrating a retention time of 10.826 min for TIM, 82% recovery efficiency, and a detection limit of 0.02 mg/kg.

### 2.5. DNA Extraction and Quantitative Analysis of Genes

Microbial DNA was isolated from compost samples collected at six time points (days 0, 3, 5, 12, 20 and 25) using the Omega E.Z.N.A.™ Soil DNA Kit (Omega Bio-tek, Norcross, GA, USA) following the standard protocol. The extracted DNA was divided into three aliquots and preserved at −20 °C for subsequent molecular analyses. A panel of fourteen target genes was examined, including eight MRGs (*erm*A, *erm*B, *erm*C, *erm*F, *erm*X, *erm*Q, *mef*A, *ere*A), two multidrug resistance genes (*acr*A, *acr*B), and four mobile genetic elements (*intI*1, *intI*2, *tnp*A, *Tn*916/1545). PCR amplification was performed in 25 μL reaction volumes containing 1 μL DNA template, 12.5 μL of 2× PCR master mix, and 0.5 μL of each specific primer. The thermal profile consisted of initial denaturation at 94 °C for 5 min, followed by 35 cycles of denaturation (94 °C, 30 s), primer-specific annealing (30 s), and extension (72 °C, 30 s), with a final extension at 72 °C for 10 min. Amplification products were verified by agarose gel electrophoresis, and target bands were excised and purified using the OMEGA Gel Extraction Kit (Omega Bio-tek, Norcross, GA, USA) for downstream applications. Quantitative assessment of gene abundance was conducted via qPCR (Bio-Rad CFX96, Bio-Rad Laboratories, Hercules, CA, USA) using the following conditions: initial denaturation at 95 °C for 2 min, then 40 cycles of 95 °C for 15 s, annealing for 30 s, and 75 °C for 30 s. Primer sequences and corresponding annealing temperatures are provided in Table S2.

### 2.6. 16S rRNA Gene High-Throughput Sequencing

Bacterial community analysis was performed through amplification of the 16S rRNA gene V3–V4 hypervariable regions using universal primers 357F (5′-ACTCCTACGGGAGGCAGCAG-3′) and 806R (5′-GGACTACHVGGGTWTCTAAT-3′). The resulting amplicons were subjected to paired-end sequencing on an Illumina MiSeq platform (Guangdong Magigene Biotechnology Co., Ltd., Guangzhou, China). Raw sequence data underwent quality control processing with Trimmomatic, followed by paired-end read assembly using FLASH software (version 1.2.11). Sequence refinement and clustering were conducted in Mothur (version 1.39.5), with operational taxonomic units (OTUs) defined at a 97% nucleotide similarity cutoff for subsequent diversity analyses.

### 2.7. Statistical Analyses

Statistical analyses were conducted using multiple software platforms, with SPSS 20.0 (IBM Corp., Armonk, NY, USA) employed for ANOVA and Duncan’s tests to evaluate treatment group differences in physicochemical properties, concentrations of TIM, abundances of MRGs, and bacterial community structure. Data visualization of TIM, MRG and MGE dynamics was conducted with Prism 8.0 (GraphPad Software, San Diego, CA, USA), while statistical analyses implemented in R 3.1.0 (R Foundation for Statistical Computing, Vienna, Austria) included microbial community diversity metrics (α- and β-diversity), tripartite correlation networks (MRG–MGE–genera), and variance decomposition modeling. Network topology illustrations were generated using Gephi 0.9.3.

## 3. Results and Discussion

### 3.1. Variation in Temperature, Moisture Content and TIM Degradation Rate During Composting

Composting temperatures rose from an initial 30 °C, reaching the thermophilic phase (>50 °C) on day 3 and maintaining it through day 12 (Figure 1C). From the thermophilic phase, temperatures decreased steadily, finally equilibrating with room temperature on day 20. Temperature serves as a critical indicator during composting, significantly regulating microbial metabolic processes and population dynamics, which ultimately determines the quality of the final compost product [25,26,27]. The moisture content of all three groups decreased gradually and stabilized during the rotting period (Figure 1B). Neither temperature nor moisture displayed statistically meaningful differences across the three experimental groups (*p* > 0.05).

The variation in TIM concentration during composting is shown in Figure 1D. Initial TIM concentrations were 529.99 ± 16.15 mg/kg DM (group H) and 246.49 ± 22.83 mg/kg DM (group L), declining to 179.20 ± 38.13 mg/kg DM and 93.44 ± 9.53 mg/kg DM by composting termination, corresponding to degradation rates of 62.09% and 66.53%, respectively. TIM degradation did not differ significantly between L and H groups (*p* > 0.05). Previous studies reported that the concentration of TIM in broiler manure decreased significantly during the composting process, with over 99% degradation observed after 40 days [11], which may differ from our findings. In previous studies, antibiotics were directly added to manure during composting [28,29,30], which may make it difficult to represent the concentration levels and physicochemical properties of the antibiotics after they are metabolized in the animal body. In our study, manure was collected from animals fed TIM-treated feed at therapeutic concentrations according to the Chinese Veterinary Pharmacopoeia. The use of manure with two doses of residual TIM after in vivo metabolism led to the results of the composting trials being more similar to those observed in practice. Additionally, the initial concentrations of TIM in the compost were as high as 529.99 mg/kg DM and 246.49 mg/kg DM, which may have contributed to the differences in TIM degradation rates. Antibiotic formulation, temperature, moisture, and processing duration emerged as pivotal parameters governing antibiotic degradation during composting [31,32,33,34], accounting for the observed discrepancies between our findings and previous studies.

### 3.2. Fate of MRGs During Manure Composting

**Figure 2:**
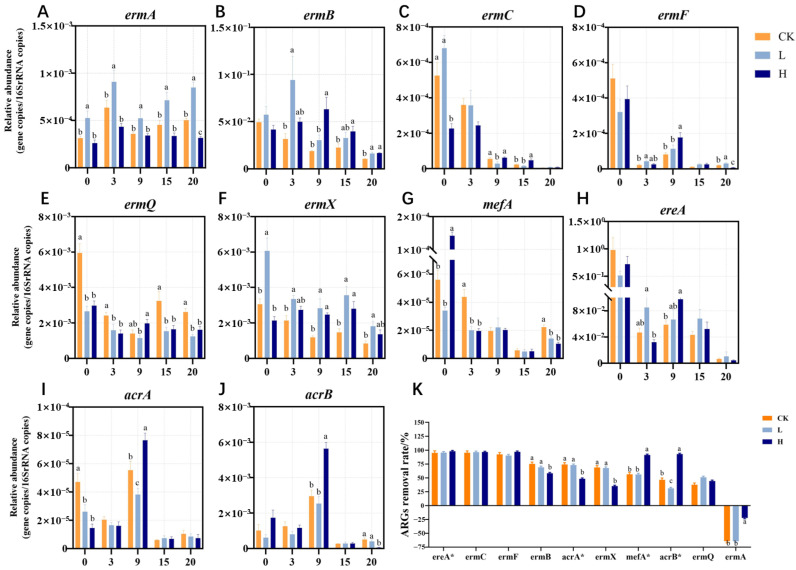
Temporal variations in relative abundance of 10 identified MRGs during composting (**A**–**J**). Removal rates of MRGs at the end of composting (**K**). The asterisk (*) in the upper right of the MRGs indicates that it is a multiple-antibiotic resistance gene. Different letters (a, b, c) denote significant inter-group differences (*p* < 0.05), while shared letters indicate non-significance. This suggests that the treatment had a significant effect on the experimental results. Groups: CK (control), L (246.49 ± 22.83 mg/kg), H (529.99 ± 16.15 mg/kg).

Eight MRGs and two multidrug resistance genes were examined in this study (Figure 2). To analyze the reasons for the different outcomes of MRGs, the targeted MRGs were classified into three categories based on their removal rates: removed, reduced, and increased. *ere*A, *erm*C, *erm*F, *mef*A (belonging to H group), and *acr*B (in H group) constituted the removed category, with removal rates exceeding 90%. In the present study, we found that the removal rates of *erm*C, *erm*F and *ere*A were greater than 90%, and these rates were not affected by antibiotic residues. These findings demonstrated a marked decrease in the relative abundances of *erm*C, *erm*F, and *ere*A genes during the thermophilic phase, likely attributable to thermal inactivation of host bacteria and plasmid degradation under elevated composting temperatures. Interestingly, the relative abundance of *ere*A clearly decreased again at 20 days (Figure 2H). The carriers of the *ere*A gene in the thermophilic stage were assumed to be thermophilic microorganisms, the abundance of which sharply decreased after the end of the thermophilic phase, resulting in a decrease in the relative abundance of *ere*A. The removal rates of *erm*B, *erm*Q, *erm*X, *mef*A (belonging to CK and L groups), *acr*A, and *acr*B (in CK and L groups) ranged from 30% to 80% (Figure 2). Although composting reduced the abundance of these MRGs, they were not effectively removed.

In our study, the abundance of *erm*A was not effectively reduced or even increased (Figure 2A). Coincidentally, Sharma et al. [35] composted cow manure produced from cattle fed tylosin or chlortetracycline–sulfamethazine and reported that the abundance of *erm*A in all the treatments increased unexpectedly from weeks 5 to 11 after an initial reduction from weeks 0 to 5. Additionally, we observed that the increase in *erm*A was significantly greater in the CK and L groups than in the H group. In our study, composting with no antibiotics or low concentrations of TIM residues promoted the enrichment of *erm*A, whereas high concentrations of TIM residues inhibited this increase. This phenomenon may be related to the status and activity of MGEs and their host bacteria.

### 3.3. Variations in MGEs During Composting

**Figure 3:**
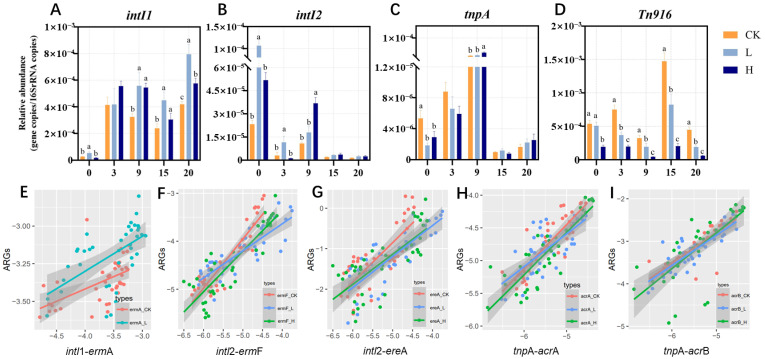
Dynamics of MGEs (*intI*1, *intI*2, *tnp*A and *Tn*916/1545) during composting (**A**–**D**). Different letters (a, b, c) denote significant inter-group differences (*p* < 0.05), while shared letters indicate non-significance. Groups: CK (control), L (246.49 ± 22.83 mg/kg), H (529.99 ± 16.15 mg/kg). Correlations between the relative abundances of MRGs and MGEs (**E**–**I**).

Correlation analysis between MRGs and MGEs is presented in Figure 3A–D. Extensive research has demonstrated that HGT mediated by MGEs represents a primary mechanism for ARG dissemination among bacterial populations [36]. In this study, final *intI*1 levels were significantly elevated versus initial concentrations in all treatments (Figure 3A, *p* < 0.05), revealing composting’s limited capacity for *intI*1 reduction. This finding aligns with established literature reports [37,38]. Additionally, a positive correlation was observed between *intI*1 and *erm*A in the CK and L groups (Figure 3E, *p* < 0.05), which indicated that *intI*1 and *erm*A may be linked in a gene cassette and that transmission occurs under low-TIM stress. This may be an important cause of *erm*A rebound in the later stages of composting. In contrast, under high concentrations of TIM residues, susceptible bacteria and even host bacteria carrying some resistance genes were inhibited or killed, leading to the absence of a correlation between *erm*A and *intI*1 in the H group. High concentrations of antibiotics inhibit bacterial growth, while the process of conjugative transfer is also suppressed. These findings suggest that low therapeutic doses of TIM residues may promote HGT mediated by *intI*1, thereby increasing the abundance of *erm*A during composting. Conjugative transfer is the process by which bacteria exchange genetic material through a conjugation pilus. This process typically relies on bacterial growth and active metabolism. At high concentrations, antibiotics inhibit bacterial growth or induce cell death, thereby indirectly affecting the transfer of plasmids or other MGEs [39]. However, sub-minimal inhibitory concentrations (sub-MICs) of antibiotics have been shown to significantly promote HGT by increasing the conjugation frequency. For example, sub-MIC levels of meropenem, ciprofloxacin, cefotaxime, and amikacin were found to upregulate the expression of conjugation-related genes and enhance plasmid-mediated transfer in *Klebsiella pneumoniae* and *Escherichia coli* both in vitro and in vivo [40]. The environmental concentrations of tetracycline (ranging from 3.9 to 250 ng/mL) increased horizontal gene transfer from the donor *Vibrio parahaemolyticus* NJIFDCVp52 to the recipient *Escherichia coli* EC600 through the mobilizable plasmid pVP52-1, with an increase of 1.47- to 3.19-fold. The underlying mechanism may involve the promotion of genes associated with conjugative transfer in both the donor and recipient cells by tetracycline, as well as the overproduction of reactive oxygen species and increased cell membrane permeability [41].

*IntI*2 abundance showed 93.76% (CK), 97.66% (L), and 95.30% (H) reductions by composting termination. *erm*F and *ere*A abundances positively correlated with *IntI*2 levels (*p* < 0.05), implying their co-removal during composting. Cao et al. [42] reported that the addition of a composite microbial agent in pig manure composting promoted a reduction in total ARG levels. The decrease in the abundance of MGEs such as *IntI*2 and *Tn*916/1545 was identified as a key factor contributing to the reduction in macrolide and tetracycline resistance genes in the compost. The abundances of *acr*A and *acr*B were positively correlated with the abundance of *tnp*A (*p* < 0.05). During the maturation phase, the decrease in *tnp*A abundance may be the primary cause of the reduction in *acr*A and *acr*B abundance.

### 3.4. Changes in Microbial Diversity and Microbial Community Structure During Composting

**Figure 4:**
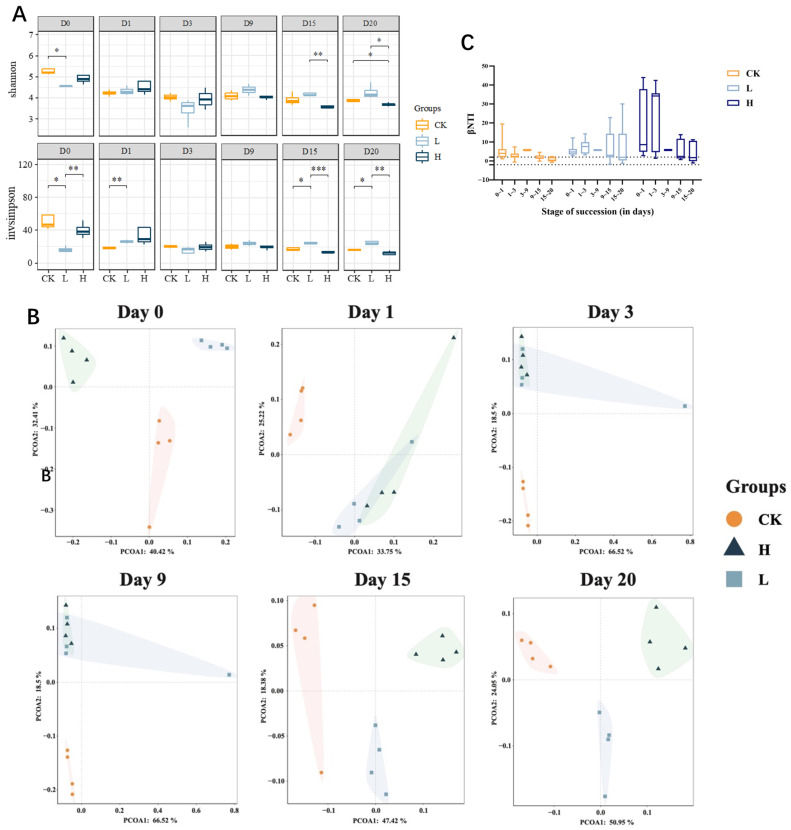
Microbial diversity dynamics across composting treatments. Alpha diversity dynamics (Shannon/Simpson) during composting (**A**). PCoA (Bray–Curtis) of bacterial community succession during composting (95% C.I. ellipses) (**B**). Data points are colored and shaped by treatment group: orange circles for CK, light blue squares for L, and dark blue triangles for H. Each subplot corresponds to a sampling time point from D0 to D20. The light-colored shaded areas surrounding each group of points represent 95% confidence ellipses, which visualize the distribution range of samples within each group and highlight the dissimilarities between groups. The weighted abundance β-average nearest classification distance (**C**). Asterisks indicate statistical significance: * for *p* < 0.05, ** for *p* < 0.01, and *** for *p* < 0.001. Groups: CK (control), L (246.49 ± 22.83 mg/kg), H (529.99 ± 16.15 mg/kg).

The Shannon index reflects species diversity, whereas the Simpson index indicates species richness. During the composting process, the α diversity of the CK and H groups gradually decreased and then stabilized (Figure 4A). In contrast, the α diversity of the L group remained relatively stable throughout the composting process. By the end of composting, the α diversity in the L group was greater than that in the CK and H groups. These findings suggest that composting significantly reduces the α diversity of microbial communities, whereas low concentrations of TIM residues can mitigate the impact of composting on microbial α diversity, thereby maintaining community stability.

The impact of TIM residues on microbial β diversity during composting is shown in Figure 4B. Initial composting stages showed large distances among CK, L, and H groups, indicating that TIM feeding affected the β diversity of microbial communities in pig manure. As composting progressed into the heating and high-temperature phases (days 1–9), the sample distances between the L and H groups gradually decreased, whereas the distances between the CK group and the L and H groups remained relatively large. This suggests that during the high-temperature phase, the presence of TIM residues had a more pronounced effect in altering the bacterial community structure than the dose of TIM. During the maturation phase (days 15–20), the distances among the CK, L, and H groups increased, and the microbial community structures differed significantly among the groups. This finding indicates that the dose-dependent effect of TIM residues significantly influenced the bacterial community structure during the late stage of composting.

We used the weighted abundance β-mean nearest taxon distance (β MNTD) to quantify the phylogenetic turnover of the microbial communities. We used a null model to generate expected β MNTD values to infer the ecological factors driving the observed turnover in community assemblages, given the dominant influence of stochastic processes. To evaluate the magnitude and direction of the deviation between the observed β MNTD values and the null β MNTD distribution, we calculated the β-nearest taxon index (β NTI) (Figure 4C). A β NTI value of <−2 or >+2 indicates that the observed phylogenetic turnover is significantly lower or higher than expected, respectively. Significant deviations (|β NTI| > 2) suggest that a deterministic process is the dominant factor, whereas smaller deviations (|β NTI| < 2) indicate that stochastic processes prevail [43]. In our study, we observed that the |β NTI| values in the CK group were predominantly greater than 2 during the heating and thermophilic phases, gradually decreased through the maturation phase, and ultimately decreased below 2 by the end of composting. These findings suggest that microbial turnover in the CK group was predominantly deterministic during the heating and thermophilic phases, whereas it became more stochastic during the maturation phase. High temperatures during the thermophilic phase likely led to the elimination of sensitive microbes and suppressed the growth of certain taxa, thereby driving community succession in a deterministic direction. In contrast, as temperatures decreased during the maturation phase, conditions became more favorable for microbial growth, allowing the revival of many taxa, which resulted in a shift toward stochastic processes in community succession. In the L group, the |β NTI| values remained consistently above 2 for most of the composting process, with some values falling below 2 toward the end of the composting period. The residual low levels of TIM in the L group inhibited certain microbial taxa, leading community succession to be primarily deterministic. However, as TIM was degraded during the later stages of composting, stochastic processes began to emerge in community turnover. Notably, during the initial and heating phases, the |β NTI| values in the H group were significantly greater than those in the CK and L groups. This can be attributed to the higher concentration of TIM residues in the H group, which eliminated a substantial proportion of sensitive microbes, leaving behind a community dominated by heat-tolerant, antibiotic-resistant taxa, thereby driving community turnover in a highly deterministic manner. As the composting process progressed, with decreasing temperatures and TIM degradation, the deterministic influence weakened, and stochastic processes began to emerge. Therefore, throughout the composting process, high temperatures and antibiotic residues predominantly drove community turnover in a deterministic manner. However, as the process progressed, the decline in temperature and the degradation of TIM reduced the deterministic influence, allowing stochastic processes to gradually become more pronounced.

### 3.5. Microbial Taxonomic Shifts at Phylum and Genus Levels During Composting

**Figure 5:**
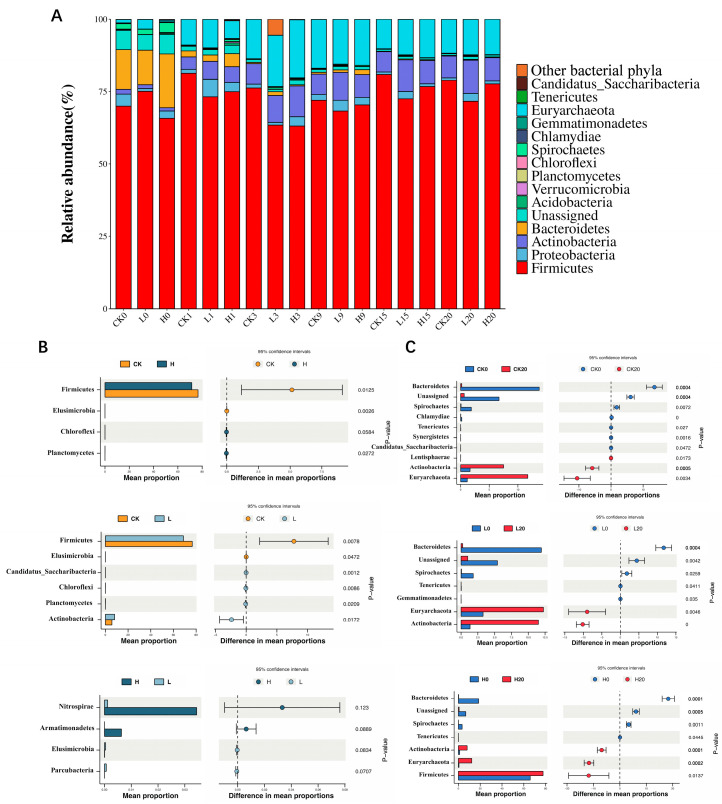
Phylum-level community dynamics across composting groups (**A**). Differentially abundant microorganisms at the phylum level (**B**,**C**). Groups: CK (control), L (246.49 ± 22.83 mg/kg), H (529.99 ± 16.15 mg/kg).

Figure 5A shows that the dominant phyla in the composting process were Firmicutes, Euryarchaeota, Actinobacteria, Bacteroidetes, and Proteobacteria. The relative abundances of Firmicutes, Euryarchaeota, Actinobacteria, Bacteroidetes and Proteobacteria in all the treatment groups decreased first but then increased during the composting process. On day 0 of composting, the relative abundance of Firmicutes in each treatment group ranged from 65.84% to 75.08%, with no significant difference among the three treatment groups (*p* < 0.05). There was no significant difference in the relative abundance of Euryarchaeota, Actinobacteria, Bacteroidetes, or Proteobacteria among these three groups (*p* > 0.05). Figure 5B shows that during the whole composting process, the relative abundances of Firmicutes and Euryarchaeota in the CK group were significantly greater than those in the L group and H group (*p* < 0.05), and the relative abundance of Euryarchaeota in the L group was significantly greater than that in the H group (*p* < 0.05). The relative abundances of Chloroflexi and Planctomycetes in the L and H groups were significantly greater than those in the CK group. Our results indicated that the relative abundances of Firmicutes and Euryarchaeota were significantly reduced by TIM residues and that higher concentrations of TIM residues had greater inhibitory effects on the relative abundance of Euryarchaeota. The TIM residues significantly increased the relative abundances of Chloroflexi and Planctomycetes in the composting system. Figure 5C reveals stable Firmicutes abundance in all groups from day 0 to day 20 of composting (*p* > 0.05). The relative abundances of Bacteroidetes, Spirochaetes and Tenericutes in all groups decreased significantly, whereas the relative abundances of Actinobacteria and Euryarchaeota increased significantly.

In the differential analysis of the top ten genera with the highest abundance among all treatments over the composting cycle (Figure S3), the relative abundances of Streptococcus, Eubacterium, and Olsenella were significantly greater in the CK group than in the L and H groups (*p* < 0.05). Conversely, the relative abundances of *Enterococcus* and *Methanosphaera* were significantly greater in the L and H groups than in the CK group (*p* < 0.05). Furthermore, the relative abundance of *Streptococcus* in the L group was significantly greater than that in the H group (*p* < 0.05). In the differential analysis of microbial community composition at the genus level during composting (as illustrated in the Supplementary Materials), a significant decline in the relative abundances of *Roseburia* and *Intestinibacter* was noted in all treatments by composting termination versus the initial phase. Conversely, the relative abundances of *Lactobacillus*, *Streptococcus*, *Methanobrevibacter*, and *Corynebacterium* significantly increased across the three groups. These results indicate that the composting treatment significantly reduced the relative abundances of *Roseburia* and *Intestinibacter* while markedly increasing the relative abundances of *Lactobacillus*, *Streptococcus*, *Methanobrevibacter*, and *Corynebacterium* in pig manure.

The observed shifts in microbial community structure and composition induced by TIM residues, as evidenced by the significant β-diversity separation and the alteration of dominant phyla, prompt a deeper inquiry into the nature of this selective pressure. While the classical definition of a prebiotic—a substrate that selectively stimulates the growth of beneficial microorganisms [44]—is fundamentally at odds with the antimicrobial nature of TIM, our results intriguingly suggest that TIM exerts a powerful prebiotic-like selective force on the bacterial population. Here, however, “selection” operates through a starkly different mechanism: the compound does not serve as a nutrient but as a stressor that selectively inhibits susceptible taxa, thereby vacating ecological niches and conferring a competitive advantage to resistant bacteria [45]. This process ultimately drives the deterministic succession of the community, favoring the proliferation of antibiotic-resistant populations and the horizontal gene transfer of ARGs like *erm*A, as mediated by *intI1*. Consequently, the “selection” imposed by TIM is unintentional and paradoxical, ultimately promoting the enrichment and dissemination of resistance determinants, which poses a potential environmental risk. This stands in direct contrast to the goal of a true prebiotic, which is to improve ecosystem health by specifically fostering beneficial taxa. This framework of TIM acting as a selective agent, rather than a nutritive one, provides a crucial context for interpreting the subsequent network co-occurrence patterns between MRGs and bacterial taxa.

### 3.6. Relationship Between Microbial Taxa and MRGs

**Figure 6:**
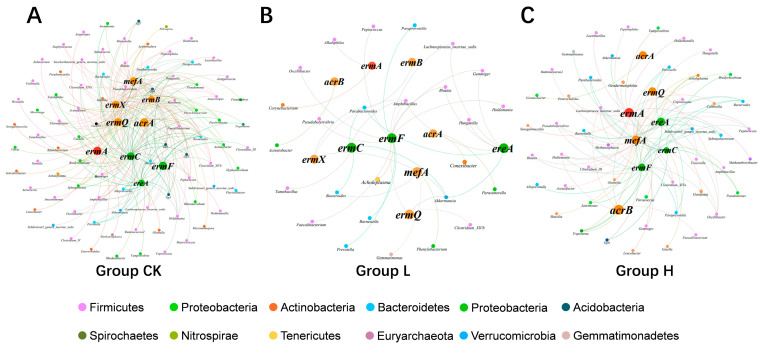
Co-occurrence network linking ten MRGs to dominant host genera (abundance > 0.1%) (correlation > 0.6, *p* < 0.05). In the network, nodes represent the following: (1) green nodes—MRGs with >90% removal efficiency; (2) orange nodes—MRGs with 30–70% removal efficiency; and (3) red nodes—MRGs showing increased abundance. The edges represent significant co-occurrence relationships between MRGs and their potential microbial hosts. (**A**) represents the co-occurrence network of the CK group, (**B**) represents that of the L group, and (**C**) represents that of the H group.

To investigate potential symbiotic associations between bacterial communities and MRGs, network analysis was performed using dominant bacterial genera (relative abundance > 0.1%) and MRGs across all composting phases. Network analysis revealed distinct topological features across treatment groups, with CK, L, and H groups containing 188/249, 72/39, and 114/93 nodes/edges, respectively (Figure 6). These results indicate that TIMs (especially in the L group) diminished putative MRG–host bacterial diversity and network connectivity. In these two groups (CK/H), significant correlations were observed between MRGs and their potential host bacteria, indicating that horizontal gene transfer of resistance genes was limited. These findings suggest that composting can effectively reduce MRGs by targeting and eliminating these putative MRG vectors. Contrastingly, the L group exhibited the lowest MRG–host bacterial diversity yet the highest terminal MRG abundance. We speculate that the low-dose concentration of TIM may have facilitated the HGT of MRGs over the composting cycle, thereby contributing to the rebound of specific MRGs, such as *erm*A. A significant link was observed between ARG levels and microbial community dynamics, suggesting the occurrence of horizontal gene transfer in this setting [46]. Furthermore, the potential host bacteria for MRGs in the CK group were Firmicutes (39.36%), Proteobacteria (17.02%), Bacteroidetes (10.64%), and others. In the L group, the main host bacteria for MRGs were Firmicutes (39.36%), Bacteroidetes (13.89%), Proteobacteria (8.33%), and others. In the H group, the predominant host bacteria for MRGs were Firmicutes (33.33%), Actinobacteria (15.79%), Bacteroidetes (12.28%), and others. These results indicate that Firmicutes is the primary potential host for MRGs across all treatment groups.

*Faecalibacterium*, *Holdemania*, *Alloprevotella*, and *Barnesiella* were identified as common hosts of *ere*A, *erm*C, and *erm*F in the CK group. These bacterial hosts may play crucial roles in the removal of *ere*A, *erm*C, and *erm*F in this group. In contrast, *Lachnospiracea_incertae_sedis* emerged as the predominant host for these MRGs in the H group. These results suggest that, under relatively high concentrations of TIM, *Lachnospiracea_incertae_sedis* may have played a pivotal role in the elimination of MRGs in the treatment group. In the CK group, the potential hosts responsible for the reduction in the levels of various MRGs (*acr*A, *acr*B, *erm*B, *erm*X, *erm*Q, and *mef*A) were primarily *Gemmiger*, *Faecalibacterium*, and *Phenylobacterium*. In the L group, the main bacterial hosts associated with reduced MRGs were *Parabacteroides*, *Akkermansia*, and *Parabacteroides*. In the H group, the key potential hosts for the reduced MRGs included *Lachnospiracea_incertae_sedis*, *Subdivision 5 genus incertae_sedis*, and *Clostridium III*. These bacteria reduced the abundances of multiple MRGs and may have been the key microbial players driving the reduction in MRGs abundance in each treatment group.

### 3.7. Key Microbial Genera and Their Contributions to Physicochemical Changes During Composting

**Figure 7:**
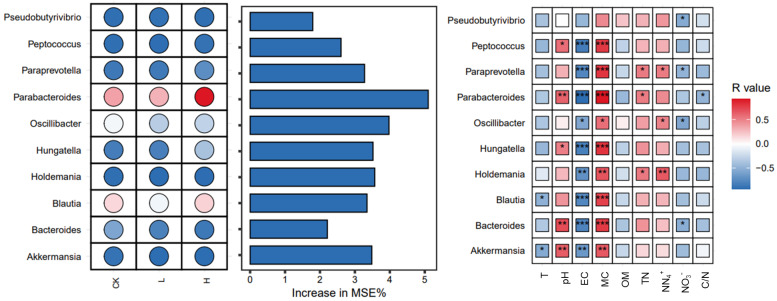
Random forest search for biomarkers and environmental drivers. In the left panel, color depth (saturation) indicates the relative importance of bacteria in each group (CK, L, H), with darker shades representing higher importance. Blue and red hues denote abundance levels: blue indicates lower abundance or negative correlation, while red indicates higher abundance or positive correlation. Asterisks indicate statistical significance: * for *p* < 0.05, ** for *p* < 0.01, and *** for *p* < 0.001. Groups: CK (control), L (246.49 ± 22.83 mg/kg), H (529.99 ± 16.15 mg/kg). T, temperature. EC, electrical conductivity. OM, organic matter. TN, total nitrogen. ${\mathrm{NO}}_{3}^{-}$, nitrate nitrogen. ${\mathrm{NH}}_{4}^{+}$, ammonium nitrogen.

Composting is a complex process involving the decomposition of organic matter, which is mediated by diverse microbial communities. These microbes play essential roles in driving physicochemical transformations, such as temperature regulation, organic matter degradation, and nitrogen cycling [47,48,49]. Exploring key microbial genera and their relationships with environmental factors can help elucidate their roles and optimize composting efficiency. We employed a combination of microbial abundance analysis, random forest modeling, and correlation analysis to identify significant genera and their ecological functions under different treatment conditions [50]. The importance of microbial taxa in distinguishing between groups was evaluated using a random forest model, with the increase in the mean squared error (MSE%) serving as the metric (Figure 7). Among the top 10 genera associated with MRG hosts, *Parabacteroides* was identified as the most significant, underscoring its critical role during the composting process. Statistical analysis revealed that the *Parabacteroides* abundance was significantly positively correlated with pH and total nitrogen (TN) and highly positively correlated with moisture content. Additionally, Parabacteroides was identified as the host for several MRGs, including *erm*B, *erm*C, and *ere*A. These MRGs were effectively eliminated or reduced during the composting process, suggesting that Parabacteroides may play a pivotal role in MRGs attenuation. Among the environmental factors, moisture content is likely the primary driver influencing the activity and abundance of Parabacteroides. Moreover, the analysis demonstrated that most microbial biomarkers exhibited highly significant correlations with moisture content and electrical conductivity (EC). These findings highlight the essential role of these environmental parameters in shaping microbial activity during composting. The results emphasize that optimizing moisture content and EC could enhance microbial processes and improve the efficiency of MRG removal, offering valuable insights for advancing composting management strategies.

## 4. Conclusions

This study comprehensively evaluated the effects of composting on the degradation of TIM antibiotic residues and the fate of macrolide resistance genes (MRGs) in pig manure, with a focus on the role of initial residue concentrations (high vs. low).

Our key findings demonstrate that the composting process effectively elevated temperatures to a thermophilic phase and reduced moisture, yet the degradation rate of TIM (approximately 62–66%) was lower than some previously reported values. This discrepancy is likely attributable to the use of in vivo-metabolized manure, which presents antibiotic residues in forms that may be more recalcitrant or complexed with organic matter, thus offering a more realistic simulation of field conditions. Notably, the initial TIM concentration did not significantly influence its own degradation efficiency. More critically, composting induced complex dynamics in the MRG profiles. While genes such as *ere*A, *erm*C, and *erm*F were effectively removed (>90%), others like *erm*B, *erm*Q, and *erm*X were only partially reduced (30–80%). A particularly important finding was the significant rebound of *erm*A in groups with no or low antibiotic residues (CK and L), an effect that was suppressed in the high-concentration group (H). This suggests a concentration-dependent hormetic effect, where sub-inhibitory levels of TIM may inadvertently promote the selection and horizontal gene transfer of certain resistance determinants. Network and correlation analyses further identified that the rebound of *erm*A was closely linked to the integron *intI1* under low-TIM stress, highlighting the critical role of MGEs in facilitating the persistence and spread of antibiotic resistance in composting ecosystems. Furthermore, microbial community analysis revealed that TIM residues, particularly at high concentrations, acted as a strong deterministic selective pressure, significantly altering β-diversity and enriching potential antibiotic-resistant hosts within phyla like Firmicutes and Actinobacteria.

This study offers clear guidance for managing antibiotic-containing manure: composting is recommended as a viable process for reducing antibiotic residues and a broad spectrum of resistance genes, yet it requires monitoring due to its gene-specific outcomes, necessitating checks for persistent genes like *erm*A; ultimately, source reduction remains the key solution, emphasizing the need to minimize antibiotic use in livestock husbandry to prevent the entry of these contaminants into the compost system.

## Data Availability

The original contributions presented in this study are included in the article. Further inquiries can be directed to the corresponding authors.

## Supplementary Materials

The following supporting information can be downloaded at https://www.mdpi.com/article/10.3390/microorganisms13092123/s1. Table S1: Properties of compost raw materials; Table S2: Primers and annealing temperature of PCR/qPCR; Figure S1: Physicochemical properties of composts from different group; Figure S2: Total relative abundance of all MRGs at the end of composting; Figure S3: Changes in microbial communities at the genus level in the three groups during composting and differential microorganisms at the genus level [12,51,52,53,54,55,56,57,58,59].

## Abbreviations

The following abbreviations are used in this manuscript:

ARGs	Antibiotic resistance genes
EC	Electrical conductivity
HGT	Horizontal gene transfer
MGEs	Mobile genetic element
MIC	Minimal inhibitory concentration
β MNTD	β-mean nearest taxon distance
MRGs	Macrolide resistance genes
MSE	Mean squared error
${\mathrm{NH}}_{4}^{+}$-N	Ammonium nitrogen
${\mathrm{NO}}_{3}^{-}$-N	Nitrate nitrogen
β NTI	β-nearest taxon index
OM	Organic matter
OTU	Operational taxonomic unit
T	Temperature
TIM	Tilmicosin
TN	Total nitrogen
TOC	Total organic carbon
VGT	Vertical gene transfer
