# Supplementary material for: Dose-Dependent Effect of Tilmicosin Residues on ermA Rebound Mediated by IntI1 in Pig Manure Compost
Source: Microorganisms. 2025 Sep 11;13(9):2123. doi: 10.3390/microorganisms13092123 (PMC12472723; doi:10.3390/microorganisms13092123)
Supplement: Supplementary file 1 [file microorganisms-13-02123-s001.zip › microorganisms-3840114-supplementary.pdf]

Supplemental Materials

Dose-effect of tilmicosin residues on *erm*A rebound mediated by *IntI*1 in pig manure compost

Captions

Table S1 Properties of Compost Raw Materials

Table S2 Primers and annealing temperature of PCR/qPCR.

Fig. S1 Physicochemical properties of composts from different groups.

Fig. S2 Total relative abundance of all MRGs at the end of composting.

Fig. S3. Changes in microbial communities at the genus level in the three groups during composting and differential microorganisms at the genus level.

Table S1 Properties of Compost Raw Materials

Raw material	Water content (%)	TOC (g/kg DM)	TN (g/kg DM)	C/N
Swine manure in CK group	70.09 ± 0.11	401.92 ± 6.09	29.89 ± 1.64	13.47 ± 0.81
Swine manure in L group	70.11 ± 0.03	408.36 ± 12.00	30.64 ± 1.75	13.36 ± 0.88
Swine manure in H group	70.91 ± 0.03	409.58 ± 5.98	28.74 ± 1.43	14.28 ± 0.91
Sawdust	7.26 ± 0.64	483.98 ± 1.36	1.09 ± 0.13	423.57 ± 91.71

Table S2 Primers and annealing temperature of PCR/qPCR.

Genes	Primer sequence (5'-3')	Annealing temperature (℃)	Size (bp)	Reference
16S rRNA	F:GTGSTGCAYGGYTGTCGTCA<br>R:ACGTCRTCCMCACCTTCCTC	60	146	[1]
*erm*A	F:GGTTTGCTATTGATGGTGGAA<br>R:GAACGCGATATTCACGGTTTA	55	190	[2]
*erm*B	F:AAAACTTACCCGCCATACCA<br>R:TTTGGCGTGTTTCATTGCTT	55	139	[2]
*erm*C	F:AATCGTCAATTCCTGCATGT<br>R:TAATCGTGGAATACGGGTTTG	55	299	[3]
*erm*F	F:CGACACAGCTTTGGTTGAAC<br>R:GGACCTACCTCATAGACAAG	56	309	[4]
*erm*X	F: GAGATCGGRCCAGGAAGC<br>R: GTGTGCACCATCGCCTGA	60	488	[5]
*erm*Q	F:CACCAACTGATATGTGGCTAG<br>R:CTAGGCATGGGATGGAAGTC	60	154	[6]
*mph*A	F:GTGAGGAGGAGCTTCGCGAG<br>R:TGCCGCAGGACTCGGAGGTC	60	403	[7]
*mef*A	F:AGTATCATTAATCACTAGTGC<br>R:TTCTTCTGGTACTAAAAGTGG	60	348	[8]
*ere*A	F:ACTCGTATATGGCGGGCGTAGTAG<br>R:GGCGTGGTCACAACAGAGAATCC	60	257	Self-designed
*acr*A	F:CGGTCGTTCTGATGCTCT<br>R:GATGTCGCTACCTTCTTTGA	60	230	Self-designed
*acr*B	F:AACTACGACATCATCGCAGAG<br>R:GCGTCATCCACCAACAGG	60	407	Self-designed
*int*I1	F:CCTCCCGCACGATGATC	55	280	[9]

	R:TCCACGCATCGTCAGGC			
*int*I2	F:GTTATTTTATTGCTGGGATTAGGC<br>R:TTTTACGCTGCTGTATGGTGC	57	164	[10]
*tnp*A	F:CGCTTTGTTACGCCAGTC<br>R:TTCAGCACGCCATAGTCG	60	344	Self-designed
*Tn*916/1545	F:GACAGTATTAAGCCATCAGAC<br>R:TCTTCCGAACACAATCATCT	50	142	Self-designed

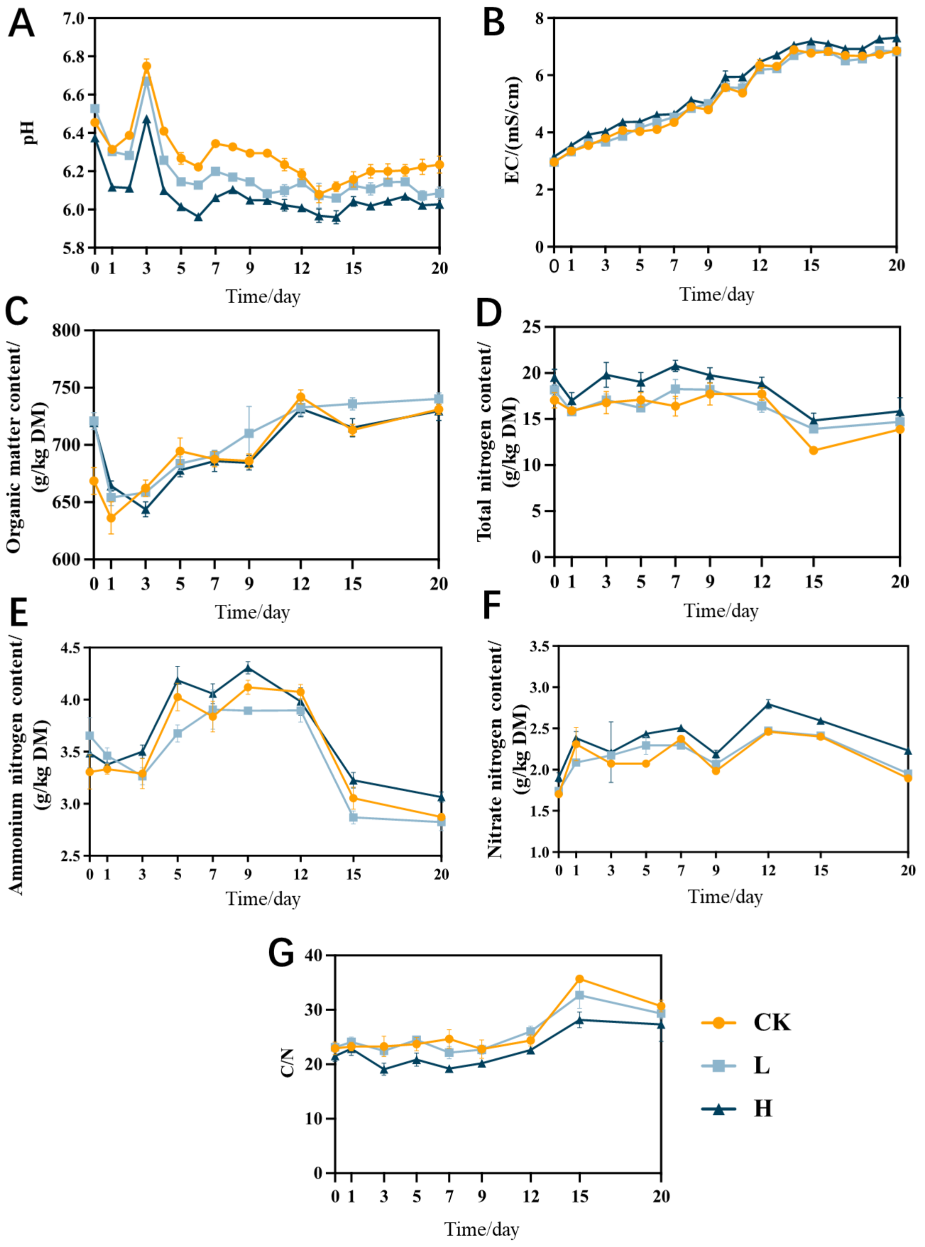

Figure S1 Physicochemical properties of composts from different groups. The physicochemical properties include pH (A), electrical conductivity (B), organic matter content (C), total nitrogen content (D), ammonium nitrogen content (E), nitrate nitrogen (F) and C/N ratio (G). CK: control group; L: low concentration (246.49±22.83 mg/kg) TIM group; H: high concentration (529.99±16.15 mg/kg) TIM group.

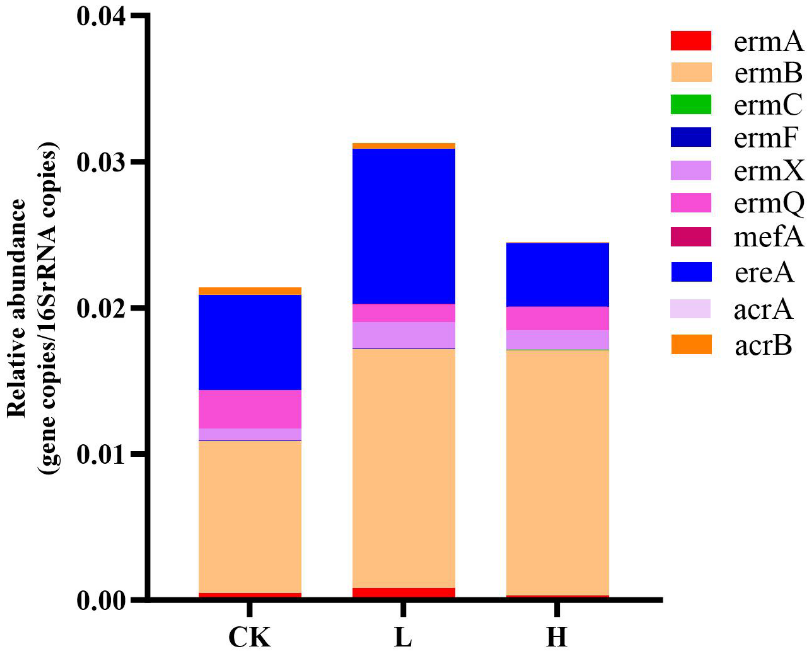

Fig. S2 Total relative abundance of all MRGs at the end of composting. CK: control group; L: low concentration (246.49±22.83 mg/kg) TIM group; H: high concentration (529.99±16.15 mg/kg) TIM group.

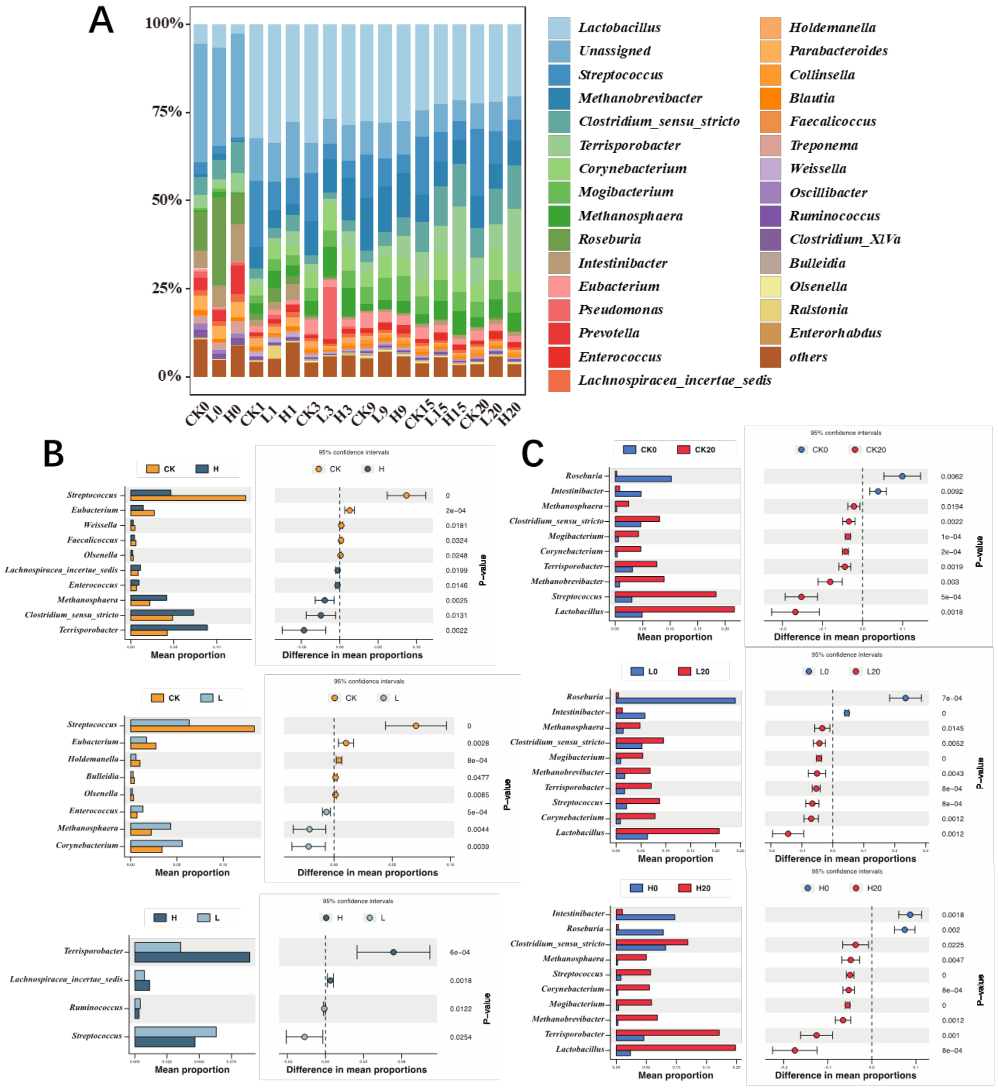

Fig. S3. Changes in microbial communities at the genus level in the three groups during composting (A). Differential microorganisms at the genus level (B and C). CK: control group; L: low concentration (246.49±22.83 mg/kg) TIM group; H: high concentration (529.99±16.15 mg/kg) TIM group.

References:

1. Rafraf, I.D.; Lekunberri, I.; Sànchez-Melsió, A.; Aouni, M.; Borrego, C.M.; Balcázar, J.L. Abundance of antibiotic resistance genes in five municipal wastewater treatment plants in the Monastir Governorate, Tunisia. *Environ. Pollut.* **2016**, 219, 353-358.
2. Kristiansson, E.; Fick, J.; Janzon, A.; Grabic, R.; Rutgersson, C.; Weijdegård, B.; Söderström, H.; Larsson, D.J. Pyrosequencing of antibiotic-contaminated river sediments reveals high levels of resistance and gene transfer elements. *PLoS ONE* **2011**, 6(2), e17038.
3. Zhou, H.; Wang, X.; Li, Z.; Kuang, Y.; Mao, D.; Luo, Y. Occurrence and distribution of urban dust-associated bacterial antibiotic resistance in Northern China. *Environ. Sci. Technol. Lett.* **2018**, 5(2), 50-55.
4. Chen, J.; Yu, Z.; Michel Jr, F.C.; Wittum, T.; Morrison, M. Development and application of real-time PCR assays for quantification of erm genes conferring resistance to macrolides-lincosamides-streptogramin B in livestock manure and manure management systems. *Appl. Environ. Microbiol.* **2007**, 73(14), 4407-4416.
5. Klima, C.; Alexander, T.; Read, R.; Gow, S.; Booker, C.; Hannon, S.; Sheedy, C.; McAllister, T.; Selinger, L. Genetic characterization and antimicrobial susceptibility of Mannheimia haemolytica isolated from the nasopharynx of feedlot cattle. *Vet. Microbiol.* **2011**, 149(3-4), 390-398.
6. Mu, Q.; Li, J.; Sun, Y.; Mao, D.; Wang, Q.; Luo, Y. Occurrence of sulfonamide-, tetracycline-, plasmid-mediated quinolone-and macrolide-resistance genes in livestock feedlots in Northern China. *Environ. Sci. Pollut. Res. Int.* **2015**, 22, 6932-6940.
7. Liu, Y.; Li, H.; Lv, N.; Zhang, Y.; Xu, X.; Ye, Y.; Gao, Y.; Li, J. Prevalence of plasmid-mediated determinants with decreased susceptibility to azithromycin among Shigella isolates in anhui, China. *Front. Microbiol.* **2020**, 11, 1181.
8. Sutcliffe, J.; Grebe, T.; Tait-Kamradt, A.; Wondrack, L. Detection of erythromycin-resistant determinants by PCR. *Antimicrob. Agents Chemother.* **1996**, 40(11), 2562-2566.
9. Xu, L.; Chen, H.; Canales, M.; Ciric, L. Use of synthesized double-stranded gene fragments as qPCR standards for the quantification of antibiotic resistance genes. *J. Microbiol. Methods* **2019**, 164, 105670.
10. Liao, H.; Lu, X.; Rensing, C.; Friman, V.P.; Geisen, S.; Chen, Z.; Yu, Z.; Wei, Z.; Zhou, S.; Zhu, Y. Hyperthermophilic composting accelerates the removal of antibiotic resistance genes and mobile genetic elements in sewage sludge. *Environ. Sci. Technol.* **2018**, 52(1), 266-276.
